# The Applicability of TaqMan-Based Quantitative Real-Time PCR Assays for Detecting and Enumerating *Cryptosporidium* spp. Oocysts in the Environment

**DOI:** 10.1371/journal.pone.0066562

**Published:** 2013-06-21

**Authors:** Sarah E. Staggs, Erin M. Beckman, Scott P. Keely, Reena Mackwan, Michael W. Ware, Alan P. Moyer, James A. Ferretti, Abu Sayed, Lihua Xiao, Eric N. Villegas

**Affiliations:** 1 National Exposure Research Laboratory, US Environmental Protection Agency, Cincinnati, Ohio, United States of America; 2 Dynamac Corporation, Cincinnati, Ohio, United States of America; 3 Department of Biological Sciences, McMicken School of Arts and Sciences, University of Cincinnati, Cincinnati, Ohio, United States of America; 4 Region 2, US Environmental Protection Agency, Edison, New Jersey, United States of America; 5 Centers for Disease Control and Prevention, Atlanta, Georgia, United States of America; Royal Tropical Institute, The Netherlands

## Abstract

Quantitative real-time polymerase chain reaction (qPCR) assays to detect *Cryptosporidium* oocysts in clinical samples are increasingly being used to diagnose human cryptosporidiosis, but a parallel approach for detecting and identifying *Cryptosporidium* oocyst contamination in surface water sources has yet to be established for current drinking water quality monitoring practices. It has been proposed that *Cryptosporidium* qPCR-based assays could be used as viable alternatives to current microscopic-based detection methods to quantify levels of oocysts in drinking water sources; however, data on specificity, analytical sensitivity, and the ability to accurately quantify low levels of oocysts are limited. The purpose of this study was to provide a comprehensive evaluation of TaqMan-based qPCR assays, which were developed for either clinical or environmental investigations, for detecting *Cryptosporidium* oocyst contamination in water. Ten different qPCR assays, six previously published and four developed in this study were analyzed for specificity and analytical sensitivity. Specificity varied between all ten assays, and in one particular assay, which targeted the *Cryptosporidium 18S rRNA* gene, successfully detected all *Cryptosporidium* spp. tested, but also cross-amplified *T. gondii*, fungi, algae, and dinoflagellates. When evaluating the analytical sensitivity of these qPCR assays, results showed that eight of the assays could reliably detect ten flow-sorted oocysts in reagent water or environmental matrix. This study revealed that while a qPCR-based detection assay can be useful for detecting and differentiating different *Cryptosporidium* species in environmental samples, it cannot accurately measure low levels of oocysts that are typically found in drinking water sources.

## Introduction

*Cryptosporidium*, the causative agent of cryptosporidiosis, is an intracellular apicomplexan parasite that infects the gastrointestinal tracts of animals and humans. Infection typically occurs through the fecal-oral route with waterborne transmission being most common. The disease is a self-limiting gastroenteritis but can potentially be fatal in immunocompromised individuals [1]. Effective treatment of cryptosporidiosis remains a challenge due to the lack of efficacious drugs [2]. Currently, 26 *Cryptosporidium* species have been identified with dozens more genotypes reported [3]–[9]. Of the known species and genotypes reported, at least 12 have been found to infect humans, with *Cryptosporidium parvum* and *Cryptosporidium hominis* being associated with over 90% of the cryptosporidiosis outbreaks [5]–[7], [10], [11].

In the United States alone over 58,000 cases of waterborne associated cryptosporidiosis occurred between 1995 and 2007 and in 1993, over 400,000 cases of cryptosporidiosis were reported during the Milwaukee outbreak [12], [13]. Contamination of the drinking water supplies with *Cryptosporidium* has also been reported. More than 80% of surface water supplies and over 25% of treated drinking waters in the United States were reported to be contaminated with oocysts, underscoring the human health risks associated with this pathogen [14]. In an effort to improve human health and reduce risks posed by *Cryptosporidium* in drinking water systems, the United States Environmental Protection Agency (USEPA) promulgated the Long Term 2 Enhanced Surface Water Treatment Rule, which requires public drinking water utilities to monitor surface source waters for the presence of *Cryptosporidium* oocysts using standardized methods such as USEPA Method 1622 [15]. This rule also serves to supplement existing regulations by employing additional treatments to higher risk systems.

USEPA Method 1622 uses a microscope-based detection method to enumerate oocysts in the environment. Although this method has been proven useful in monitoring wastewater [16] and drinking water matrices [17], [18], it has certain limitations which include the inability to differentiate between species or determine infectivity of the detected oocysts. Thus, methods that are more user-friendly, less labor intensive, and faster at identifying, genotyping, and determining oocyst viability are currently being explored for use in surveillance monitoring practices within the water industry [17], [19]–[22].

Quantitative real-time PCR has proven itself to be a more user-friendly and faster approach to detect and enumerate microorganisms in various environmental samples [23], making it a promising candidate for detecting waterborne pathogens such as *Cryptosporidium*. Indeed, a *Cryptosporidium*-specific qPCR assay has been proposed as a technique for detecting *Cryptosporidium* oocysts in water matrices [24]–[27]. As with many molecular detection assays, there are several potential limitations associated with this technique including its range of specificity and ability to detect a single oocyst in environmental matrices. Since recent studies have shown levels of *Cryptosporidium* oocysts found in surface drinking water sources to be very low along with its relatively low infectious dose, it is critical that any methods proposed should have reliable detection limits down to one oocyst [14], [28]. While there have also been many reports of qPCR-based *Cryptosporidium* detection assays for clinical investigations [25], [29]–[32], their use for detecting a single *Cryptosporidium* oocyst in environmental samples, like raw surface waters, as alternatives to the microscopic enumeration component of USEPA Method 1622 have not been thoroughly assessed.

The objective of this study was to determine the applicability of a TaqMan-based qPCR assay for detecting *C. hominis*, *C. parvum*, or all *Cryptosporidium* spp. oocysts in water matrices. Specificity and analytical sensitivity of ten qPCR assays either published in the literature or newly developed in-house were determined. More importantly, the ability of these assays to reliably detect and resolve low levels of oocysts (e.g., 1, 2, and 5, vs. 10 oocysts) present in environmental matrices was investigated.

## Materials and Methods

### Ethics Statement

The use of animals in this study was approved and carried out in strict accordance with the recommendations by the US Environmental Protection Agency’s Institutional Animal Care and Use Committee. All efforts were made to minimize animal suffering (USEPA-CI IACUC: 08–10).

### *Cryptosporidium* Oocysts

*C. parvum* oocysts (Harley Moon-Iowa isolate) and *Cryptosporidium muris* oocysts (RN66) were purchased from Waterborne, Inc. (New Orleans, LA, USA). *C. parvum* oocysts were propagated using immunosuppressed CF-1 mice as described [33]. Oocysts were purified from feces by sieving, step sucrose gradients, and cesium chloride purification [34]. Purified oocysts were then resuspended in reagent grade water containing 100 U/ml penicillin, 100 µg/ml streptomycin, and 0.25 µg/ml amphotericin B (Life Technologies, Gaithersburg, MD, USA) and stored at 4°C. Purified *C. hominis* oocysts (TU502) and *Cryptosporidium meleagridis* oocysts (TU1867) were obtained from Tufts University [35], [36]. All oocysts in this study were used within three months of shed date.

### Flow Sorting of *Cryptosporidium* Oocyst Samples

A Fluorescence Activated Cell Sorter (FACS; FACS Aria II, Beckton Dickinson, Palo Alto, CA, USA) was used for enumerating oocysts as previously described [37]. Oocysts were gated by forward and side scatter (FSC and SSC, respectively), with FSC and SSC adjusted to distinctly identify the oocyst population. Oocysts ranging from 1 to 1×10^3^ were sorted in sterile 1.5 ml microcentrifuge tubes containing 100 µl of sterile reagent water and used to spike reagent grade water and source water concentrates.

### Preparation of Oocyst Spiked Environmental Samples

An Ohio River water sample with turbidity of 129 Nephelometric Turbidity Units (NTU) was collected according to USEPA Method 1622 [15]. Briefly, at least 10 L of raw water was filtered through an Envirochek HV filter (Pall, Port Washington, NY, USA). The retentate was then eluted from the filter using an elution buffer (0.01% Laureth-12 (Sigma, St. Louis, MO, USA); 10 mM Tris-Cl pH 7.4 (Sigma); 1 mM EDTA (Sigma); 0.015% Antifoam A (Sigma)) and centrifuged. Volume equivalents of 0.5 ml packed pellets were resuspended in 10 ml elution buffer and processed via an immunomagnetic separation (IMS) procedure using anti-*Cryptosporidium* coated paramagnetic beads in accordance with the manufacturer’s protocol (Invitrogen, Carlsbad, CA, USA). Following the final IMS wash step, retained beads were spiked with various numbers of flow-sorted oocysts for subsequent genomic DNA (gDNA) extraction and qPCR analysis. This was done to avoid loss during the immunomagnetic separation process and allowing for a more accurate measurement of the analytical sensitivity of the qPCR step in the presence of PCR inhibitors carried over through the IMS step of the method. The retained beads from each sample were pooled and used for all spiked environmental samples in this study. This approach increases the likelihood of capturing PCR inhibitors present in the sample as well as eliminates variability introduced when sampling at different times that have different levels of turbidity.

### Genomic DNA Extraction

Genomic DNA was extracted from *C. parvum, C. hominis, C. muris,* and *C. meleagridis* flow-sorted oocysts using the following optimized protocol adapted from the Epicentre MasterPure Complete (MPC) DNA and RNA Purification Kit (Epicentre Biotechnologies, Madison, WI, USA). Oocysts were centrifuged at 2,500×*g*, and the supernatant was removed. Oocysts were resuspended in 300 µl Tissue Cell Lysis solution (Epicentre) and subjected to five freeze/thaw cycles consisting of transferring samples between a liquid nitrogen bath and a 70°C water bath until the samples were completely frozen or thawed, respectively. Samples were then transferred into GeneRite pre-loaded glass bead tubes (GeneRite, North Brunswick, NJ, USA). Samples were bead milled twice at one minute intervals at 250 RPM using a mini-bead beater (BioSpec Products, Inc., Bartlesville, OK, USA). Samples were placed on ice in between the bead milling steps to avoid excessive heat buildup during the process. Next, 50 µg of Proteinase K (Epicentre) was added to each tube, and the samples were incubated for 1 hr at 56°C with constant agitation. Following digestion, samples were centrifuged at 21,000×*g* for 1 min, and supernatants were transferred to nuclease-free microcentrifuge tubes. Five micrograms of RNase A (Epicentre) was added to each tube, and samples were incubated for 30 min at 37°C. Following incubation, 150 µl of MPC Protein Precipitation solution (Epicentre) was added to each sample and then centrifuged at room temperature for 10 min at 12,000×*g*. Supernatants were transferred into new nuclease-free microcentrifuge tubes and 500 µl of isopropanol (Sigma) was added to each tube. The tubes were inverted at least 30 times and placed on ice for 30 min. DNA was pelleted by centrifugation at 4°C for 15 min at 21,000×*g*. DNA pellets were washed twice with 500 µl of 75% ethanol and then allowed to dry at room temperature before resuspension in 100 µl nuclease-free water.

Genomic DNA samples used for specificity testing were obtained from the following sources. *Cryptosporidium canis* and *Cryptosporidium felis* DNA were extracted from environmental samples as previously described [17]. *Bacillus thuringiensis* (35646-D), *Bacillus cereus* (10987-D), *Shigella flexneri* (29903-D), and *Escherichia coli K-12* (10798-D) gDNA were purchased from American Type Culture Collection (ATCC, Manassas, VA, USA). Genomic DNA from *Giardia muris*, *Giardia duodenalis* (H3; Assemblage B), *Encephalitozoon hellem* (CDC:0291:V213), *Encephalitozoon intestinalis* (50502, ATCC), and *Encephalitozoon cuniculi* (50502, ATCC) were isolated from cysts/spores propagated at the USEPA animal facilities or from cell cultures as previously described [38]–[40], and DNA was extracted using the Epicentre kit extraction procedure described above. *Schistosoma mansoni* gDNA was kindly provided by Dr. Robert Greenberg (Department of Pathobiology, University of Pennsylvania, School of Veterinary Medicine, Philadelphia, PA, USA). *Toxoplasma gondii* gDNA was isolated from the VEG strain (oocysts provided by J.P. Dubey, US Department of Agriculture, Beltsville, MD, USA) using the modified Epicentre kit extraction procedure described above.

### Quantitative Real-time PCR

Primers to detect *Cryptosporidium* spp. using a TaqMan-based qPCR assay are listed in Table 1. All forward and reverse primers, JVA probe, JVAG1 probe, and JVAG2 probe were obtained from Integrated DNA Technologies (Coralville, IA, USA). The CRU18S probe, CRULib13Cp probe, and CRULib13Ch probe were designed as minor grove binding (MGB) probes and obtained from Applied Biosystems (Carlsbad, CA, USA). The qPCR reaction consisted of 40 cycles of template denaturation at 95°C for 15 sec, with an annealing temperature of 60°C for 1 min. For the Cp001and Ch001 qPCR assays, the annealing temperature was adjusted to 64°C for 50 sec. QPCRs consisted of 4 µl of template DNA into a total reaction volume of 25 µl. Each reaction contained 1×PCR Buffer II, 4.2 mM MgCl_2_, 240 µM dNTP mix, 1×ROX reference dye, 1.5 U of AmpliTaq Gold polymerase (Applied Biosystems), 240 nM forward and reverse primers, and 120 nM probe. To minimize the effects of PCR inhibitors present in each sample, 400 ng/µl non-acetylated bovine serum albumin (Sigma) was also added to the qPCR reactions [41]. An ABI PRISM® 7000 Sequence Detection System (Applied Biosystems) was used for all qPCR analysis unless otherwise stated. Triplicate qPCR reactions were performed for each sample analyzed.

**Table 1:** Primer and probe sequences used in this study.

Target organism	Name	Target gene	primer/probe	Sequence (5′→3′)	References
*C. hominis*	Ch001	DNA-J like	Forward	ATC CTA CGT CTA ACT TCA CGC	This study
			Reverse	GAA GCT CTT CAT ATG CCT TAT TA	
			Probe^1^	TTA TAG GGA TAC CAG TCG ATT CTG ATG AC	
	Ch003	NTF2	Forward	AGC ATT ATT GGA TGT TGT AG	This study
			Reverse	TAA TAA AAA TCA ATC AGT GGT	
			Probe^1^	CTG CTA ATA CTA GTA ATA CTA GTA GTA ATC	
	CRULib13Ch^2^	Unknown	Forward	TCC TTG AAA TGA ATA TTT GTG ACT CG	[30]
			Reverse	AAA TGT GGT AGT TGC GGT TGA AA	
			Probe^3^	CTT ACT TCG TGG CGG CGT	
	JVAG1^4^	Unknown	Forward	ACT TTT TGT TTG TTT TAC GCC G	[29]
			Reverse	AAT GTG GTA GTT GCG GTT GAA	
			Probe^1^	ATT TAT TAA TTT ATC TCT TAC TTC GT	
*C. parvum*	Cp001	DNA-J like	Forward	ATC CTA CGT CTA ACT TCA CGT	This study
			Reverse	GTA GCT CCT CAT ATG CCT TAT TG	
			Probe^1^	TTA TAG GGA TAC CAG TCG ATT CTG ATG AT	
	Cp003	NTF2	Forward	AGC ATT ATT GGA TGT TGT AT TAG	This study
			Reverse	TAA TAA AAG TCA ATC AGT GGC	
			Probe^1^	CTG CTA ATA CTA GTA ATA ATA GTA GTA ATA	
	CRULib13Cp^2^	Unknown	Forward	TCC TTG AAA TGA ATA TTT GTG ACT CG	[30]
			Reverse	TTA ATG TGG TAG TTG CGG TTG AAC	
			Probe^3^	TAT CTC TTC GTA GCG GCG TA	
	JVAG2^4^	Unknown	Forward	ACT TTT TGT TTG TTT TAC GCC G	[29]
			Reverse	AAT GTG GTA GTT GCG GTT GAA	
			Probe^1^	ATT TAT CTC TTC GTA GCG GCG	
*Cryptosporidium* spp.	CRU18S	18S rRNA	Forward	GAG GTA GTG ACA AGA AAT AAC AAT ACA GG	[30]
			Reverse	CTG CTT TAA GCA CTC TAA TTT TCT CAA AG	
			Probe^5^	TAC GAG CTT TTT AAC TGC AAC AA	
	JVA	18S rRNA	Forward	ATG ACG GGT AAC GGG GAA T	[24]
			Reverse	CCA ATT ACA AAA CCA AAA AGT CC	
			Probe^1^	CGC GCC TGC TGC CTT CCT TAG ATG	

1 Probes labeled with 6-carboxy-fluorescein (FAM) and black hole quencher (BHQ).

2 Forward primer is the same.

3 Probes labeled with VIC and with minor groove binding non-fluorescent quencher (MGB).

4 Forward and reverse primers are the same.

5 Probes labeled with 6-carboxy-fluorescein (FAM) and with minor groove binding non-fluorescent quencher (MGB).

### DNA Sequencing of Environmental Samples

PCR amplicons were cloned into the plasmid vector pCR4-TOPO (Invitrogen) according to manufacturer’s protocols. Sequenced PCR products were first purified using the QIAquick PCR Purification kit according to manufacturer’s protocols (Qiagen, Valencia, CA, USA). DNA sequences of the inserts were determined by utilizing the ABI PRISM Big Dye Terminator cycle sequencing kit (Applied Biosystems) and an ABI Prism 3730xl DNA Analyzer (Applied Biosystems). Sequences were submitted to NCBI (accession numbers: JX471018-JX471053).

### Statistical and Bioinformatic Analyses

Statistical analysis was performed using Sigma Plot 11 (Systat Software, Inc. San Jose, CA, USA) or Excel 2007 (Microsoft, Redmond, WA, USA). Unless otherwise stated, a non-parametric Mann-Whitney rank sum test was used to determine statistical significance among the different conditions tested. *P* values less than 0.05 were considered significant. DNA sequences were identified by comparison to the NCBI BLASTn database [42]. Primers were aligned to a comprehensive list of *18S rRNA* sequences with TestProbe implemented on the website http://arb-silva.de [43]. PCR primers and probes were aligned to *Cryptosporidium* reference *18S rRNA* sequences with CAP implemented in BioEdit [44]. The relationships of environmental DNA sequences were inferred using the Neighbor-Joining method [45]. Trees were inferred with Molecular Evolutionary Genetics Analysis, version 5 (MEGA5) [46] using the Jukes-Cantor substitution model [47]. Bootstrap values were computed as described [48].

## Results

### Identification and Development of *Cryptosporidium* Genus- and Species-specific qPCR Assays

In order to establish a qPCR assay that could potentially detect all *Cryptosporidium* species and genotypes, two primer/probe sets were initially identified from the literature, JVA [24], [29] and CRU18S [30], both originally designed for clinical investigations targeting the multi-copy *18S rRNA* gene of *Cryptosporidium*. It was reported that the JVA primer sets could detect at least ten *Cryptosporidium* species: *C. hominis, C. parvum, C. canis, C. felis, C. ubiquitum, C. muris, C. andersoni, C. baileyi, C. serpentis, and C. wrairi.* In this study, the JVA qPCR assay detected *C. parvum*, *C. hominis*, *C. meleagridis*, and *C. felis* but not *C. muris* or *C. canis* (Table 2). Additionally, the JVA qPCR assay did not detect any other genera tested, which was consistent with previous studies. As for the CRU18S qPCR assay, it detected all of the *Cryptosporidium* species tested in the current study (Table 2). Moreover, Hadfield and colleagues reported that the CRU18S set also detected several other *Cryptosporidium* species and genotypes including *C. cuniculus*, *C. andersoni*, *C. felis*, *C. ubiquitum*, *C. bovis*, *C. baileyi*, *C. canis*, *C. xiaoi*, and the *Cryptosporidium* horse genotype [30]. Specificity analyses of the CRU18S assay revealed that while no qPCR signals were detected from distantly related organisms including *S. mansoni*, *E. coli, B. thuringiensis, B. cereus, S. flexneri, E. hellem, E. intestinalis, E. cuniculi*, or closely related species like *G. muris* and *G. duodenalis*, significant qPCR signals were detected with *T. gondii* gDNA (1×10^3^ oocysts; C_T_ values ∼30). This was different than the results reported by Hadfield and colleagues, where no cross-reactivity with *T. gondii* was observed. Triplicate qPCR reactions were performed for each specificity test. Sequence alignment analyses of the JVA primers and probes indicated that the forward primer and probe sequences were identical to many *Cryptosporidium spp*. as well as sequences from *Eimeria tenella*, *Cyclospora cayetanensis*, and *T. gondii* (Figure S1). Specificity for this qPCR assay was partially achieved by designing the reverse primer to span one of the hypervariable regions in this locus. But as a consequence, it may not be able to detect all *Cryptosporidium* spp. due to sequence polymorphisms found among the different species. For example, there are two nucleotide differences between the JVA reverse primer and the *18S rRNA* gene sequence in *C. muris.* A four nucleotide difference with the *C. canis 18S rRNA* gene sequence and the JVA reverse primer was found resulting in no detectable amplification signal as shown in Table 2. For CRU18S, all primers and probe sequences were highly homologous to *Cryptosporidium spp., E. tenella, C. cayetanensis*, and *T. gondii*. Overall, specificity of these *Cryptosporidium* genus-specific primer/probe sets proved to be the most variable when targeting the *18S rRNA* gene.

**Table 2:** QPCR results indicating positive or negative detection with a TaqMan probe and primer set^1^.

	Primer/Probe sets										
	*Cryptosporidium* spp.		*C. hominis*				*C. parvum*				
Species	CRU18S	JVA	Ch001	Ch003	JVAG1a	CRULib13 Ch	Cp001	Cp003		JVAG2a	CRULib13 Cp
**Protozoa**											
*C. parvum*	+	+	−	−	−	−	+	+		+	+
*C. hominis*	+	+	+	+	+	+	−	−		−	−
*C. meleagridis*	+	+	+	−	−	−	−		−	−	−
*C. felis*	ND^2^	+	−	−	−	ND^3^	−		−	−	ND^3^
*C. canis*	ND^2^	−	−	−	−	ND^3^	−		−	−	ND^3^
*C. muris*	+	+/−	−	−	−	−	−		−	−	−
*G. muris*	−	−	−	−	−	−	−		−	−	−
*G. duodenalis*	−	−	−	−	−	−	−		−	−	−
*T. gondii*	+^3^	−	−	−	−	−	−		−	−	−
**Bacteria**											
*B. thuringiensis*	−	−	−	−	−	−	−		−	−	−
*B. cereus*	−	−	−	−	−	−	−		−	−	−
*E. coli*	−	−	−	−	−	−	−		−	−	−
*S. flexneri*	−	−	−	−	−	−	−		−	−	−
**Fungi**											
*E. hellem*	−	−	−	−	−	−	−		−	−	−
*E. intestinalis*	−	−	−	−	−	−	−		−	−	−−
*E. cuniculi*	−	−	−	−	−	−	−		−	−	−
**Helminth**											
*S. mansoni*	ND	−	−	−	−	ND	−		−	−	ND

1 +, indicates positive qPCR signals (<40 C_T_). −, indicates negative qPCR signals. Positive qPCR results were sequence verified.

2 This primer/probe set gave positive qPCR signals for this species in Hadfield et al. [30].

3 This primer/probe set gave negative qPCR signals for this species in Hadfield et al. [30].

ND, not done.

+/−, samples had high C_T_ values close to detection limits.

Since *C. parvum* and *C. hominis* cause over 90% of the cryptosporidiosis reported worldwide [49], it is also important to establish assays to specifically detect these two species for more accurate exposure risk assessments. Therefore, four qPCR assays were tested for their ability to specifically detect *C. parvum* or *C. hominis*. Two qPCR assays identified from the literature that specifically detected *C. hominis* were the JVAG1 [29] and the CRULib13Ch [30] assays, both of which target single copy regions of unknown function. Two additional *C. hominis-*specific qPCR assays were also developed for this study, the Ch001 and Ch003, which target the DNA-J like region of the genome and a nuclear transport factor (NTF2) gene of *Cryptosporidium*, respectively (Table 1). The DNA-J like and NTF2 regions showed promise in developing species specific primer/probe sets since sequences from these two loci were different among the different *Cryptosporidium* sequences in the database. Primer/probe pairs were then designed to be specific for either *C. hominis* or *C. parvum* using BLAST (data not shown). Analysis of the *C. hominis* specific qPCR assays (Ch001, Ch003, JVAG1, and CRULib13Ch) revealed that each assay only amplified *C. hominis* DNA except the Ch001 qPCR assay, which also amplified *C. meleagridis* DNA. Sequence analysis of the Ch001 amplicon detected from the *C. meleagridis* sample aligned with *C. meleagridis* sequences deposited in NCBI (data not shown). All other non-target genomic DNA tested listed in Table 2 were not amplified by the Ch001, the Ch003, the JVAG1, or the CRULib13Ch qPCR assays. Similarly, *C. parvum* specific qPCR assays were evaluated, two from the literature, JVAG2 [29] and CRULib13Cp [30], and two developed in this study Cp001 and Cp003, which also target the DNA-J like region of the genome and a nuclear transport factor (NTF2) gene of *Cryptosporidium*, respectively. All four assays amplified *C. parvum* gDNA and did not cross react with any of the closely related coccidian species or with any of the bacteria, fungi, or helminth DNA tested (Table 2).

### Analytical Sensitivity of the *Cryptosporidium* TaqMan- based qPCR Assays

Analytical sensitivities of *Cryptosporidium* genus- and species-specific qPCR assays listed in Table 1 were evaluated. Four independent sets of gDNA extracted from both *C. parvum* and *C. hominis* flow-sorted oocysts were subjected to qPCR analysis, with each reaction performed in triplicate (Tables 3 and 4). For *C. parvum*, results indicated that JVA and CRU18S qPCR assays can easily detect 1000 flow-sorted oocysts with a 100% detection rate. At 100 oocysts, both JVA and CRU18S qPCR assays successfully detected 83% and 100% detection rates, respectively. As the numbers of oocysts decreased to 10 or less, the detection frequency also dramatically decreased for the JVA qPCR with only a 25% positive detection rate at 2 oocysts and a 0% detection rate at 1 oocyst. The CRU18S qPCR assay had a detection rate of 8% and 17% at the 2 and 1 oocyst level, respectively (Table 3).

**Table 3:** Detection of flow-sorted *C. parvum* oocysts using qPCR^1^.

	Primer/Probe set^2^				
	*Cryptosporidium* spp. specific		*C. parvum* specific		
# Oocysts	JVA	CRU18S	JVAG2	Cp003	CRULib13Cp
1000	31.26±1.16 (12/12)	32.10±1.25 (12/12)	31.74±1.48 (12/12)	35.51±0.25 (9/12)	32.58±0.30 (12/12)
100	34.65±1.65 (10/12)	35.05±0.79 (12/12)	35.78±1.44 (12/12)	39.97 (1/12)	36.22±0.95 (11/12)
10	37.03±0.92 (6/12)	38.42±0.95 (9/12)	36.61±0.85 (9/12)	*	39.06±1.23 (2/12)
5	37.37±1.28 (8/12)	39.06±0.47 (2/12)	36.36±1.01 (6/12)	*	38.40±0.01 (2/12)
2	37.64±0.84 (3/12)	38.22 (1/12)	37.08 (1/12)	*	*
1	*	39.60±0.53 (2/12)	*	*	*
0^3^	*	*	*	*	*

1 Results presented are the average ± standard deviation of four independent experiments. QPCR was performed in triplicate reactions for each experiment.

Flow-sorted oocysts spiked in reagent water were used to determine detection limits of the different qPCR assays evaluated.

2 C_T_ values ± standard deviation. Numbers in parentheses indicate the number of positive samples (C_T_ values <40) over the total reactions performed.

3 “Mock” flow-sorted sample containing no oocysts.

* , Not detected (C_T_ >40).

**Table 4:** Detection of flow-sorted *C. hominis* oocysts using qPCR^1^.

	Primer/Probe set^2^					
	*Cryptosporidium* spp. specific		*C. hominis* specific			
# Oocysts	JVA	CRU18S	JVAG1	Ch003	CRULib13Ch	
1000	30.21±0.59 (12/12)	33.65±1.47 (11/12)	33.75±1.23 (12/12)	32.33±1.54 (12/12)	35.43±1.69 (12/12)	
100	35.08±2.15 (11/12)	36.19±1.36 (9/12)	37.38±0.59 (9/12)	36.06±1.28 (9/12)	37.72±1.01 (8/12)	
10	36.62±1.48 (8/12)	37.32±1.34 (6/12)	37.25±0.71 (2/12)	37.70±0.45 (3/12)	38.23 (1/12)	
5	37.71±1.59 (5/12)	38.87±0.43 (4/12)	39.07±0.49 (2/12)	36.77±0.26 (2/12)	38.93 (1/12)	
2	36.78±1.31 (4/12)	38.10±1.10 (2/12)	*	38.31 (1/12)	38.81 (1/12)	
1	38.71±0.72 (6/12)	39.14±0.18 (2/12)	39.06 (1/12)	*	38.68 (1/12)	
0^3^	*	*	*	*	*	

1 Results presented are the average ± standard deviation of four independent experiments. QPCR was performed in triplicate reactions for each experiment.

Flow-sorted oocysts spiked in reagent water were used to determine detection limits of the different qPCR assays evaluated.

2 C_T_ values ± standard deviation. Numbers in parentheses indicate the number of positive samples (C_T_ values <40) over the total reactions performed.

3 “Mock” flow-sorted sample containing no oocysts.

* , Not detected (C_T_ >40).

Among the *C. parvum*-specific qPCR assays, JVAG2 had stronger qPCR signals as well as a higher frequency of detection when compared to CRULib13Cp or the Cp003. The JVAG2 qPCR assay was also able to detect as low as 2 oocysts (8%) while Cp003 and CRULib13Cp qPCR assays were unable to detect samples containing less than 100 and 5 oocysts, respectively. Based on these results, CRU18S was the only assay to successfully detect a single flow-sorted *C. parvum* oocyst (Table 3).

For the *C. hominis* qPCR results (Table 4), the genus-specific JVA and CRU18S assays successfully detected a single flow-sorted oocyst in reagent water, with a positive detection frequency of 50% and 17%, respectively. The species-specific qPCR assays (JVAG1, Ch003, and CRULib13Ch) had lower frequencies of detection when tested with gDNA from less than 10 oocysts. Most notable is the CRULib13Ch assay, which had a frequency of detection of 1 out of 12 replicates even when 10 oocysts were present as compared with JVA and CRU18S (67% and 50%, respectively). As for the JVAG1 and Ch003 qPCR assays, both could detect 1 and 2 oocysts, respectively, with a detection frequency of 8%. At higher oocyst spikes, the Ch003 assay had slightly better frequency of detection rate, with slightly lower C_T_ values. Another notable difference was that the JVAG1 assay was unable to detect 2 oocysts. Similar to what was observed with the *C. parvum* qPCR results, genus-specific primers had higher frequency of detection rates when compared to species-specific qPCR assays, particularly at low numbers of oocysts. It should be noted that when low numbers of oocysts are being assayed, high C_T_ values are expected, since the amount of genetic material present is close to the limit of what is detectable by qPCR. Nevertheless, an initial examination using standard curves analysis indicated that gDNA from a single oocyst was easily detected when a cut off value of 40 cycles was chosen (data not shown), which was consistent with recent studies by Caraguel and others suggesting that qPCR protocols set to do 40 cycles can theoretically yield 1×10^12^ amplicons from a single DNA molecule [50].

### Detection of *Cryptosporidium* Oocysts in Environmental Samples

To determine if PCR inhibitors present in environmental samples would affect the analytical sensitivity of the TaqMan assays, Ohio River water samples were processed using USEPA Method 1622 [15]. The resulting packed pellets were spiked with known amounts of flow-sorted oocysts and processed as described in the Materials and Methods section. Three independent sets of spiked environmental samples for both *C. parvum* and *C. hominis* were isolated, and three qPCR replicates were performed for each individual sample. Two sets of negative controls, each done in parallel with the experimental samples and in triplicate qPCR reactions, were also assayed: 1) the “0 oocyst” samples were environmental matrix containing no flow-sorted oocysts added to the sample that were processed through the IMS procedure and 2) the “unspiked” samples were reagent grade water that were also processed through IMS procedure (e.g., no oocysts and no environmental matrix added to the sample). Based on the C_T_ values, there were some slight differences in detection frequencies between the two assays. The JVA qPCR assay detected a minimum of 1 *C. parvum* oocyst with a mean C_T_ value of 38.14 (±0.48 standard deviation (SD)) with a 22% positive detection rate (Table 5). In contrast, the JVA qPCR assay was only able to detect 5 *C. hominis* oocysts with a 33% positive detection rate (Table 6). Improved detection frequencies were observed at higher *C. hominis* oocyst spike levels (100 oocysts; 67% positive detection rate). The other *Cryptosporidium* species-specific qPCR assays tested in this study, JVAG1, JVAG2, Ch003, Cp003, CRULib13Ch, and CRULib13Cp, were less consistent in detecting oocyst levels at or below 2 in environmental samples.

**Table 5:** Detection of flow-sorted *C. parvum* oocysts in environmental samples^1^.

	Primer/Probe set^2^				
	*Cryptosporidium* spp. specific		*C. parvum* specific		
# Oocysts	JVA	CRU18S	JVAG2	Cp003	CRULib13 Cp
1000	31.88±2.22 (9/9)	33.64±0.92 (9/9)	32.15±1.91 (9/9)	33.00±2.48 (9/9)	32.65±0.22 (9/9)
100	32.77±1.88 (9/9)	34.33±0.28(8/9)	32.80±2.37 (9/9)	33.50±2.64 (8/9)	34.60±0.30 (9/9)
10	37.41±1.03 (9/9)	37.66±1.47 (7/9)	37.02±0.72 (9/9)	38.01±0.99 (3/9)	37.97±1.16 (4/9)
5	38.45±0.82 (7/9)	37.14±0.68 (9/9)	37.34±1.08 (4/9)	*	38.27 (1/9)
2	38.98±0.59 (6/9)	37.26±1.14 (9/9)	*	*	38.04 (1/9)
1	38.14±0.48 (2/9)	36.61±1.10 (9/9)	38.02 (1/9)	*	*
0^3^	*	36.94±1.06 (9/9)^4^	*	*	*
Unspiked^5^	*	*	*	*	*

1 0.5 ml packed pellets from raw surface water were processed through the IMS procedure of USEPA Method 1622 and were spiked with flow-sorted *C. parvum* oocysts to determine detection limits of *Cryptosporidium* spp. and *C. parvum* specific qPCR assays on environmental samples. Results are the average C_T_ values of three independent experiments ± standard deviation. Triplicate reactions were performed for each condition tested in each of the three experiments conducted.

2 C_T_ values ± standard deviation. Numbers in parentheses are the ratio of positive qPCR reactions over the total reactions performed.

3 “Mock” flow-sorted sample containing no oocysts, environmental sample present.

4 the CRU18S pan-*Cryptosporidium* spp. cross reacted with indigenous non-*Cryptosporidium* oocyst present in the environmental sample.

5 Unspiked, sample containing no oocysts and no environmental sample.

* , Not detected (C_T_>40).

**Table 6:** Detection of flow-sorted *C. hominis* oocysts in environmental samples^1^.

	Primer/Probe set^2^				
	*Cryptosporidium* spp. specific		*C. hominis* specific		
# Oocysts	JVA	CRU18S	JVAG1	Ch003	CRULib13 Ch
1000	35.65±1.31 (9/9)	32.40±1.02 (9/9)	33.02±1.95(9/9)	33.40±1.02 (8/9)	33.76±1.61 (9/9)
100	38.46±1.22 (6/9)	33.38±0.53 (9/9)	35.94±2.15 (8/9)	36.72±0.99 (8/9)	37.98±0.96 (9/9)
10	39.58 (1/9)	31.57±1.09 (9/9)	38.91±0.29 (2/9)	38.29±0.72 (5/9)	37.51±0.42 (4/9)
5	32.56±0.22 (3/9)	31.81±0.74 (9/9)	*	38.88±0.64 (4/9)	38.36 (1/9)
2	*	32.91±0.93 (9/9)	38.41 (1/9)	37.71±0.17 (3/9)	*
1	*	32.99±0.91 (9/9)	*	39.24 (1/9)	*
0^3^	*	33.41±0.87 (9/9)^4^	*	*	*
Unspiked^5^	*	*	*	*	*

1 0.5 ml packed pellets from surface raw water were processed through the IMS procedure of USEPA Method 1622 and were spiked with flow-sorted *C. hominis* oocysts to determine detection limits of *Cryptosporidium* spp. and *C. hominis* specific qPCR assays on environmental samples. Results are the average C_T_ values of three independent experiments ± standard deviation. Triplicate reactions were performed for each condition tested in each of the three experiments conducted.

2 C_T_ values ± standard deviation. Numbers in parentheses are the ratio of positive qPCR reactions over the total reactions performed.

3 “Mock” flow-sorted sample containing no oocysts.

4 the CRU18S pan-*Cryptosporidium* spp. cross reacted with indigenous non-*Cryptosporidium* oocysts present in the environmental sample.

5 Unspiked, sample containing no oocyst and no environmental sample.

* Not detected (C_T_ >40).

Unexpectedly, the CRU18S qPCR tested positive for the 0 spike samples (no oocyst control) when environmental matrix was present, suggesting that the primers/probe set designed for this assay cross-reacted with non-*Cryptosporidium* DNA present in the environmental sample. Contamination of the assay was ruled out as the unspiked controls (IMS beads only and no environmental matrix added) and the no template qPCR controls were consistently negative (Tables 5 and 6). Sequence alignment of the CRU18S primer/probe set was conducted against several closely related coccidia (*C. parvum, C. hominis, C. canis, C. muris, C. cayetanensis, E. tenella,* and *T. gondii*) to predict any potential cross reactivity of this genus specific qPCR assay. Results revealed five mismatches between the forward and reverse primers and the targeted regions. The first two were found in the forward primer which contained a single T to C mismatch at position 6, and a single A to T mismatch (both with *T. gondii*) at position 3 relative to the 3′ end. The probe sequence was identical to all selected species aligned. The reverse primer also contained three mismatches, a G to A (with *C. cayetanensis*), an A to T (with *E. tenella* and *C. cayetanensis*), and a T to C (with *E. tenella*) at positions 10, 18 and 19 relative to the 5′ end, respectively (Figure S1).

To further identify the CRU18S DNA amplicons from the zero-spiked controls, samples were analyzed by Sanger sequencing. Extensive genetic diversity was found in the cloned amplicons (Figure S2), but *Cryptosporidium* species were not detected. BLAST analysis showed that the environmental DNA sequences were highly similar to *Basidiomycota* fungi (e.g., *Sistrotrema* or *Pachylepyrium*), green algae (e.g., *Ettlia* or *Chlamydomonas*), dinoflagellate (e.g., *Gymnodinium* or *Ornithocercus*), and amoebaflagellates (Table S1). This assay was originally intended for use in clinical samples, therefore, such cross-reactivities with these particular organisms would not typically be found in clinical samples.

### Effects of PCR Inhibitors Present in Environmental Matrices

PCR inhibitors, such as humic acid, are present in the environment and can potentially affect qPCR performance. To determine if the qPCR assays evaluated in this study were sensitive to PCR inhibitors, C_T_ values obtained from reagent water and environmental samples were compared (Tables 3, 4, 5, and 6). Samples spiked with 1000 oocysts were analyzed since it provided the largest dynamic range to detect any potential changes in C_T_ values (changes between 5–10 C_T_ values can be detected) as a consequence of PCR inhibition. Results revealed that qPCR assays that detected *C. parvum* oocysts (CRULib13Cp, JVA, and JVAG2) performed similarly in both matrices (*p*>0.50). Unexpectedly, the Cp003 qPCR assay resulted higher C_T_ values in reagent water as compared with environmental matrix (35.51±0.25 vs. 33.00±2.48, respectively, *p*<0.015). Conversely, while the effects of environmental matrix was not evident when detecting *C. hominis* oocysts using the Ch003 qPCR assay (reagent water: 32.33±1.54 vs. environmental matrix: 33.40±1.02, *p>*0.08), a reduction in signal from *C. hominis* spiked environmental samples as compared with spiked reagent water was observed when using the JVA qPCR assay (35.65±1.31 vs. 30.21±0.59, respectively, *p*<0.001). For the CRULib13Ch qPCR assay, a marked difference in C_T_ values was also detected, although the signal reduction was seen in spiked reagent water rather than with the spiked environmental sample (35.43±1.69 vs. 33.76±1.61, respectively, *p*<0.005). The CRU18S qPCR assay results were not analyzed since it cross-reacted with other species found in the environment. Overall, the effects of PCR inhibitors present in environmental samples were detected and appear to be assay dependent.

### Differentiating Low Numbers of Oocysts using qPCR

To better assess risks posed by *Cryptosporidium*, it is necessary to be able to accurately enumerate the number of oocysts contaminating the water. Both genus-specific *C. parvum* and *C. hominis* qPCR assays had higher frequencies of positive detection and decreased analytical sensitivity when compared to species-specific qPCR assays. However, the assays evaluated in this study did not have the requisite resolution to differentiate between samples containing 1, 2, 5, or 10 oocysts. For example, C_T_ values observed from 2 or 5 *C. parvum* oocysts spiked in reagent water using the JVA qPCR assay were not statistically different (37.64±0.84 SD vs. 37.37±1.28 SD, respectively, *p*>0.60) (Table 3). Differences in C_T_ values between 2 vs. 10 oocysts using the same qPCR assay were also not statistically significant (*p*>0.55). A comparison between samples spiked with a single oocyst was not possible since we could not detect any signal from any of the replicates performed. As for *C. hominis*, the JVA qPCR assay was able to differentiate samples containing 1 vs. 10 flow-sorted oocysts (*p*<0.02), however differentiating between 2, 5 or 10 oocysts was not possible (*p*>0.50). Results observed using the CRU18S qPCR assay were also similar to the JVA qPCR assay. For instance, C_T_ values detected in samples spiked with 2 or 5 *C. parvum* (Table 3) or *C. hominis* (Table 4) oocysts were not statistically different as compared with values detected in samples spiked with 10 oocysts (*p*>0.2).

## Discussion

### Specificity at the Genus and Species Level

Developing a qPCR assay that can detect all *Cryptosporidium* species as well as genotype unknown samples can be very useful for assessing total oocyst burden in the environment as well as identifying novel genotypes that can potentially cause disease in humans. One common strategy for detecting a wide range of species and genotypes is to target conserved regions of the genome, like 18S rRNA. There are many advantages for targeting the *Cryptosporidium 18S rRNA* gene: 1) it is a multi-copy gene that could theoretically increase analytical sensitivity of the PCR assay, 2) it contains conserved and polymorphic regions that have been useful towards species and genotype identification, and 3) it is currently the richest sequence database available for use to type *Cryptosporidium* spp. [10], [51]. In this study, we evaluated two qPCR assays, both of which were originally designed for use in clinical investigations and targeted the *18S rRNA* gene, for detecting multiple *Cryptosporidium* species (Table 1).

The CRU18S qPCR assay detected low numbers of oocysts from each of the major species of *Cryptosporidium* tested, but cross-amplified *T. gondii* gDNA (Table 2). Hadfield and colleagues did not detect any cross-reactivity with *T. gondii* DNA with this assay, but there are several factors that could account for this discrepancy. Although the primers and probes sequences and qPCR thermocycling conditions (with the exception that our study uses 40 cycles as opposed to 55 cycles in the Hadfield study) were the same for both assays, different master mixes and different thermocycling machine platforms were employed. This study also used gDNA isolated from freshly harvested *T. gondii* oocysts (1×10^3^), whereas Hadfield et al. used *T. gondii* reference gDNA. The observed cross-reactivity with *T. gondii* in this study was further supported by pairwise alignments of the CRU18S primers and the *18S rRNA* gene (Figure S1). There were five mismatches between the forward and reverse primers and the targeted regions. A single T to C mismatch at position 6 and a single A to T mismatch (both with *T. gondii*) at position 3 relative to the 3′ end of the forward primer. The probe sequence was identical to all selected species aligned. The reverse primer also contained three mismatches, a G to A (with *C. cayetanensis*), an A to T (with *E. tenella* and *C. cayetanensis*), and a T to C (with *E. tenella*) at positions 10, 18 and 19 relative to the 5′ end, respectively (Figure S1). A recent study showed that most single mismatches that occur at positions 3, 4, or 5 at the 3′ end of a primer can be tolerated during PCR [52]. Other sequence mismatches embedded in the middle are tolerated as well. This explains why DNA from *T. gondii*, and perhaps other coccidia, can be amplified with this qPCR assay. To further investigate the extent of CRU18S primer cross-reactivity, the primers were aligned to all eukaryotic sequences from the Silva ribosomal DNA database (version 108), which contains a comprehensive list of *16S/18S rRNA* sequences [43]. This analysis suggested that PCR cross-reactivity was likely to occur with groups other than Coccidia, such as *Piroplasmida*, *Eugregarinida*, and *Dinophyceae* as well as divisions beyond *Aveolata*, such as fungi and plants (data not shown). Indeed sequence analyses of amplicons detected in environmental samples that did not contain *Cryptosporidium* oocysts, revealed that the CRU18S qPCR assay amplified DNA from *Basidiomycota* fungi, green algae, dinoflagellates, and amoebaflagellates (Table S1). These results suggest that qPCR assays designed to amplify a more conserved region of the *18S rRNA* gene, while effective for detecting *Cryptosporidium* oocysts in clinical samples, may not be as useful for environmental monitoring. The JVA qPCR assay showed greater specificity than CRU18S in the presence of environmental matrix, but its inability to amplify *C. canis* and *C. muris* DNA in our study calls into question the genus specificity of these primers. All of the variability between the primers and primer binding sequences were observed with the reverse primer (Figure S1), JVAR was 83.3% (i.e., 1 indel and 3 mismatches) and 73.9% (i.e., 6 mismatches) identical to the primer binding sites of *C. canis* and *C. muris,* respectively.

Another strategy for monitoring *Cryptosporidium* contamination in the environment is using pathogen-specific qPCR assays. Although the eight *C. parvum/C. hominis* specific qPCR assays evaluated in this study were specific, with the exception of Ch001 (Table 2), these assays may underestimate the levels of potential pathogenic species or genotypes contaminating the water supplies. More importantly, these assays will not have the capability to detect novel species or genotypes that can cause disease in humans, like the recent identification of *C. cuniculus* or the horse and skunk genotypes [53], [54].

### Limitations of a qPCR-based Detection Assay

The JVA and CRU18S assays were the most sensitive in detecting DNA from low numbers of *C. hominis* and *C. parvum* oocysts. This is not surprising since the *18SrRNA* gene is a multi-copy gene whereas the CRULib13, JVAG, and 003 targets are single copy genes. However, the difference in C_T_ values between JVA and the JVAG qPCR assays was not as dramatic as previously reported [29]. Jothikumar and colleagues reported ten-fold lower detection limits in their species-specific assays when compared to their genus-specific assays, and there was a relative difference of ∼6 C_T_ values when comparing the two assays; such differences were not observed in this study (Tables 3 and 4). There are multiple factors that could account for the discrepancy in results. In this study, DNA was isolated from flow-sorted oocysts in water in the presence or absence of environmental factors, whereas Jothikumar and colleagues isolated DNA from serially diluted spiked stool samples. Using flow-sorted oocysts ensures the most accurate count possible, and when using serial dilutions of large numbers of oocysts or gDNA isolated from large numbers of oocysts, it is more difficult to accurately determine if the PCR assay is detecting DNA from a single oocyst. DNA isolation methods and qPCR platforms used were also different, and qPCR conditions differed between the two studies. Nonetheless, the uses of *Cryptosporidium* specific qPCR assays are certainly applicable for clinical samples where the numbers of oocysts per gram of feces are more abundant as compared with environmental samples [30]. These assays may also be of more use when testing wastewater matrices where oocyst levels are typically higher than those found in the environment.

QPCR assays evaluated in this study demonstrated that it is possible to detect low numbers of flow-sorted oocysts (2–10) in the presence of environmental factors. However, based on C_T_ values from *C. parvum* (Tables 3 and 5) and *C. hominis* (Table 4 and 6), some of the qPCR assays were sensitive to environmental-derived PCR inhibitors. While the CRULib13Cp, JVA, and JVAG2 assays used to detect *C. parvum* oocysts were not affected by environmental inhibitors, the analytical sensitivity of the JVA and Ch003 qPCR assays on detecting *C. hominis* oocysts were significantly reduced. Unexpectedly, the Cp003 and CRULib13Ch performed better in the presence of environmental matrix. The CRU18S qPCR assay was not analyzed because of the unexpected cross-reactivity to indigenous organisms present in water matrix (e.g., dinoflagellate). These findings further underscore the importance of additional internal controls that should be incorporated into all qPCR assays used for environmental monitoring [23].

Since the frequency of detection of a single oocyst is low and every sample containing a single spiked oocyst tested was not positive, the likelihood of false negatives can be high. Also, qPCR cannot definitively determine viability of the oocysts. One possibility to determine viability using molecular detection techniques would be with qPCR in conjunction with propidium monoazide (PMA) treatment. PMA is a photoreactive dye that can penetrate dead cells or damaged cells, but cannot penetrate live cells with intact membranes. PMA intercalates into the DNA and covalently binds to it upon exposure to light, therefore preventing amplification of the DNA. This method would be useful when examining mixed populations of dead and live oocysts that are likely to be found within an environmental matrix [19].

### QPCR and Compliance Monitoring

While studies that directly address limits of detection of USEPA Method 1622 have not been formally conducted, it is currently the only method that can accurately differentiate 1, 2, 3 or 5 oocyst containing samples. The qPCR assays evaluated in this study are currently not viable replacements for the microscopic enumeration component of USEPA Method 1622 for water analysis due to their limited resolution at low oocyst levels. One potential approach to more accurately quantify the number of oocysts present in a given sample containing less than 10 oocysts and potentially elucidate the true limit of detection of these qPCR assays would be the use of more sophisticated statistical approaches like a four parameter logistic curve analysis. This regression analysis was conducted using log transformed number of oocyst/PCR reactions versus the proportion of samples that were PCR positive. Preliminary results were inconclusive (data not shown), which was not surprising since this type of statistical analysis requires a much larger experimental data set. Alternatively, a Poisson distribution analysis of the fraction of positive qPCR reactions may perhaps be a more appropriate approach in estimating the average copies of amplifiable DNA in a sample, in a manner similar to a Most Probable Number (MPN) approach (Keely S., Staggs S., and Villegas E., unpublished results). Again, the use of MPN would require additional replicate sampling and may not be economically and logistically practical at this time. A more definitive approach that can potentially provide the requisite resolution to quantify and differentiate low numbers of oocyst is by digital PCR [55]; however, additional studies are needed before using this technology for monitoring microbial contamination in the environment. Nevertheless, TaqMan assays can be quite consistent, as some of the DNA extractions and qPCR experiments shown here were performed by two scientists with different levels of technical experience. Data from side-by-side DNA extractions and qPCR assay comparison revealed similar results that were not statistically significant (data not shown). Consistency among different end-users is an important factor to consider when developing any assays that will be employed by numerous people with varying levels of technical skills.

In this study, we have demonstrated several potential uses and limitations of *Cryptosporidium* genus- and species-specific molecular methods of detections. Although this approach may not be able to distinguish between very low levels of *Cryptosporidium* within a given matrix (1, 2, or 10 oocysts), it is still an important technique to include in the microbial detection toolbox since it could be used in conjunction with other *Cryptosporidium* oocyst detection methods, such as USEPA Method 1622, to provide additional information, such as species or genotype. The additional species/genotype information will be valuable towards developing more accurate risk assessment models associated with human health and waterborne exposure to microbial pathogens.

## Supporting Information

Figure S1 **ClustalW alignment of select 18S rRNA sequences with JVA and CRU18S qPCR primers/probes.** Species abbreviations: Cpa, *Cryptosporidium parvum*; Cho, *Cryptosporidium hominis*; Cca, *Cryptosporidium canis*; Cmu, *Cryptosporidium muris*; Cyc, *Cyclospora cayetanensis*; Ete, *Eimeria tenella*; Tgo, *Toxoplasma gondii*. Letters highlighted in grey indicate nucleotide mismatches with the CRU18S primer/probe set.
(DOCX)

Figure S2 **Relationships of 36 environmental sequences to reference** ***Cryptosporidium*** **18S rRNA sequences.** The percentage (>50) of replicate trees in which the associated taxa clustered together in the bootstrap test (500 replicates) are shown next to the branches. The Neighbor-Joining tree is drawn to scale, with branch lengths in the same units as those of the Jukes-Cantor distances used to infer the tree. Branches labeled with 1-G or 2-G prefixes are environmental samples. (JX471018-JX471053).
(DOCX)

Table S1 **BLAST analyses of the 36 environmental sequences obtained from using the CRU18S qPCR primer set^1^.**
(DOCX)

## References

[1] Fayer R (2004) *Cryptosporidium*: a water-borne zoonotic parasite. Vet Parasitol 126: 37–56. 15567578 10.1016/j.vetpar.2004.09.004

[2] Rossignol J-F (2010) *Cryptosporidium* and *Giardia*: Treatment options and prospects for new drugs. Experimental Parasitology 124: 45–53. 19632225 10.1016/j.exppara.2009.07.005

[3] Morgan-Ryan UM, Fall A, Ward LA, Hijjawi N, Sulaiman I, et al (2002) *Cryptosporidium hominis* n. sp. (Apicomplexa: Cryptosporidiidae) from *Homo sapiens* . J Eukaryot Microbiol 49: 433–440. 12503676 10.1111/j.1550-7408.2002.tb00224.x

[4] Fayer R, Santin M (2009) *Cryptosporidium xiaoi* n. sp. (Apicomplexa: Cryptosporidiidae) in sheep (*Ovis aries*). Vet Parasitol 164: 192–200. 19501967 10.1016/j.vetpar.2009.05.011

[5] Fayer R, Santin M, Macarisin D (2010) *Cryptosporidium ubiquitum* n. sp. in animals and humans. Vet Parasitol 172: 23–32. 20537798 10.1016/j.vetpar.2010.04.028

[6] Robinson G, Wright S, Elwin K, Hadfield SJ, Katzer F, et al (2010) Re-description of *Cryptosporidium cuniculus* Inman and Takeuchi, 1979 (Apicomplexa: Cryptosporidiidae): morphology, biology and phylogeny. Int J Parasitol 40: 1539–1548. 20600069 10.1016/j.ijpara.2010.05.010

[7] Fayer R (2010) Taxonomy and species delimitation in *Cryptosporidium* . Exp Parasitol 124: 90–97. 19303009 10.1016/j.exppara.2009.03.005

[8] Smith HV, Nichols RA (2010) *Cryptosporidium*: detection in water and food. Exp Parasitol 124: 61–79. 19501088 10.1016/j.exppara.2009.05.014

[9] Ren X, Zhao J, Zhang L, Ning C, Jian F, et al (2012) *Cryptosporidium tyzzeri* n. sp. (Apicomplexa: Cryptosporidiidae) in domestic mice (*Mus musculus*). Exp Parasitol 130: 274–281. 21803038 10.1016/j.exppara.2011.07.012

[10] Xiao L (2009) Molecular epidemiology of cryptosporidiosis: An update. Exp Parasitol. 10.1016/j.exppara.2009.03.018 19358845

[11] Yang R, Fenwick S, Potter A, Ng J, Ryan U (2011) Identification of novel *Cryptosporidium* genotypes in kangaroos from Western Australia. Vet Parasitol 179: 22–27. 21402448 10.1016/j.vetpar.2011.02.011

[12] MacKenzie WR, Kazmierczak JJ, Davis JP (1995) An outbreak of cryptosporidiosis associated with a resort swimming pool. Epidemiol Infect 115: 545–553. 8557087 10.1017/s0950268800058714 PMC2271606

[13] Yoder JS, Beach MJ (2010) *Cryptosporidium* surveillance and risk factors in the United States. Exp Parasitol 124: 31–39. 19786022 10.1016/j.exppara.2009.09.020

[14] Smith HV, Rose JB (1998) Waterborne cryptosporidiosis: current status. Parasitol Today 14: 14–22. 17040684 10.1016/s0169-4758(97)01150-2

[15] USEPA (2005) Method 1622: *Cryptosporidium* in water by filtration/IMS/FA. Office of Water EPA 815-R-05-001.

[16] Quintero-Betancourt W, Gennaccaro AL, Scott TM, Rose JB (2003) Assessment of methods for detection of infectious *Cryptosporidium* oocysts and *Giardia* cysts in reclaimed effluents. Appl Environ Microbiol 69: 5380–5388. 12957926 10.1128/AEM.69.9.5380-5388.2003 PMC194950

[17] Yang W, Chen P, Villegas EN, Landy RB, Kanetsky C, et al (2008) *Cryptosporidium* source tracking in the Potomac River watershed. Appl Environ Microbiol 74: 6495–6504. 18776033 10.1128/AEM.01345-08 PMC2576682

[18] Jiang J, Alderisio KA, Xiao L (2005) Distribution of *Cryptosporidium* genotypes in storm event water samples from three watersheds in New York. Appl Environ Microbiol 71: 4446–4454. 16085835 10.1128/AEM.71.8.4446-4454.2005 PMC1183313

[19] Brescia CC, Griffin SM, Ware MW, Varughese EA, Egorov AI, et al (2009) *Cryptosporidium* Propidium Monoazide-PCR, a Molecular Biology-Based Technique for Genotyping Viable *Cryptosporidium* Oocysts. Appl Environ Microbiol 75: 6856–6863. 19749067 10.1128/AEM.00540-09 PMC2772443

[20] Di Giovanni GD, LeChevallier MW (2005) Quantitative-PCR assessment of *Cryptosporidium parvum* cell culture infection. Appl Environ Microbiol 71: 1495–1500. 15746352 10.1128/AEM.71.3.1495-1500.2005 PMC1065146

[21] Johnson AM, Di Giovanni GD, Rochelle PA (2011) Comparing Assays for Sensitive and Reproducible Detection of Cell Culture-Infectious *Cryptosporidium parvum* and *Cryptosporidium hominis* in Drinking Water. Appl Environ Microbiol. 10.1128/AEM.06444-11 PMC3255618 22038611

[22] Ryu H, Tran H, Ware MW, Iker B, Griffin S, et al (2011) Application of leftover sample material from waterborne protozoa monitoring for the molecular detection of Bacteroidales and fecal source tracking markers. J Microbiol Methods 86: 337–343. 21693138 10.1016/j.mimet.2011.06.001

[23] Haugland RA, Siefring SC, Wymer LJ, Brenner KP, Dufour AP (2005) Comparison of Enterococcus measurements in freshwater at two recreational beaches by quantitative polymerase chain reaction and membrane filter culture analysis. Water Res 39: 559–568. 15707628 10.1016/j.watres.2004.11.011

[24] Hill VR, Kahler AM, Jothikumar N, Johnson TB, Hahn D, et al (2007) Multistate evaluation of an ultrafiltration-based procedure for simultaneous recovery of enteric microbes in 100-liter tap water samples. Appl Environ Microbiol 73: 4218–4225. 17483281 10.1128/AEM.02713-06 PMC1932788

[25] Fontaine M, Guillot E (2002) Development of a TaqMan quantitative PCR assay specific for *Cryptosporidium parvum* . FEMS Microbiol Lett 214: 13–17. 12204366 10.1111/j.1574-6968.2002.tb11318.x

[26] Fontaine M, Guillot E (2003) An immunomagnetic separation-real-time PCR method for quantification of *Cryptosporidium parvum* in water samples. J Microbiol Methods 54: 29–36. 12732419 10.1016/s0167-7012(03)00005-8

[27] Masago Y, Oguma K, Katayama H, Ohgaki S (2006) Quantification and genotyping of *Cryptosporidium* spp. in river water by quenching probe PCR and denaturing gradient gel electrophoresis. Water Sci Technol 54: 119–126. 10.2166/wst.2006.457 17037142

[28] Messner MJ, Chappell CL, Okhuysen PC (2001) Risk assessment for *Cryptosporidium*: a hierarchical Bayesian analysis of human dose response data. Water Res 35: 3934–3940. 12230176 10.1016/s0043-1354(01)00119-1

[29] Jothikumar N, da Silva AJ, Moura I, Qvarnstrom Y, Hill VR (2008) Detection and differentiation of *Cryptosporidium hominis* and *Cryptosporidium parvum* by dual TaqMan assays. J Med Microbiol 57: 1099–1105. 18719179 10.1099/jmm.0.2008/001461-0

[30] Hadfield SJ, Robinson G, Elwin K, Chalmers RM (2011) Detection and differentiation of *Cryptosporidium* spp. in human clinical samples by use of real-time PCR. J Clin Microbiol 49: 918–924. 21177904 10.1128/JCM.01733-10 PMC3067739

[31] Haque R, Roy S, Siddique A, Mondal U, Rahman SM, et al (2007) Multiplex real-time PCR assay for detection of *Entamoeba histolytica*, *Giardia intestinalis*, and *Cryptosporidium* spp. Am J Trop Med Hyg 76: 713–717. 17426176

[32] Stroup SE, Roy S, McHele J, Maro V, Ntabaguzi S, et al (2006) Real-time PCR detection and speciation of *Cryptosporidium* infection using Scorpion probes. J Med Microbiol 55: 1217–1222. 16914651 10.1099/jmm.0.46678-0

[33] Ware MW, Villegas EN (2010) Improved *Cryptosporidium parvum* oocyst propagation using dexamethasone suppressed CF-1 mice. Vet Parasitol 168: 329–331. 20036060 10.1016/j.vetpar.2009.11.019

[34] Arrowood MJ, Donaldson K (1996) Improved purification methods for calf-derived *Cryptosporidium parvum* oocysts using discontinuous sucrose and cesium chloride gradients. J Eukaryot Microbiol 43: 89S. 8822880 10.1111/j.1550-7408.1996.tb05015.x

[35] Akiyoshi DE, Feng X, Buckholt MA, Widmer G, Tzipori S (2002) Genetic analysis of a *Cryptosporidium parvum* human genotype 1 isolate passaged through different host species. Infect Immun 70: 5670–5675. 12228296 10.1128/IAI.70.10.5670-5675.2002 PMC128364

[36] Akiyoshi DE, Dilo J, Pearson C, Chapman S, Tumwine J, et al (2003) Characterization of *Cryptosporidium meleagridis* of human origin passaged through different host species. Infect Immun 71: 1828–1832. 12654797 10.1128/IAI.71.4.1828-1832.2003 PMC152090

[37] Ware MW, Wymer L, Lindquist HD, Schaefer FW 3rd (2003) Evaluation of an alternative IMS dissociation procedure for use with Method 1622: detection of *Cryptosporidium* in water. J Microbiol Methods 55: 575–583. 14607401 10.1016/j.mimet.2003.06.001

[38] Hester JD, Varma M, Bobst AM, Ware MW, Lindquist HD, et al (2002) Species-specific detection of three human-pathogenic microsporidial species from the genus Encephalitozoon via fluorogenic 5′ nuclease PCR assays. Mol Cell Probes 16: 435–444. 12490145 10.1006/mcpr.2002.0442

[39] Hayes SL, Rice EW, Ware MW, Schaefer FW 3rd (2003) Low pressure ultraviolet studies for inactivation of *Giardia muris* cysts. J Appl Microbiol 94: 54–59. 12492923 10.1046/j.1365-2672.2003.01805.x

[40] Schaefer FW 3rd, Johnson CH, Hsu CH, Rice EW (1991) Determination of *Giardia lamblia* cyst infective dose for the Mongolian gerbil (*Meriones unguiculatus*). Appl Environ Microbiol 57: 2408–2409. 1768111 10.1128/aem.57.8.2408-2409.1991 PMC183585

[41] Jiang J, Alderisio KA, Singh A, Xiao L (2005) Development of procedures for direct extraction of *Cryptosporidium* DNA from water concentrates and for relief of PCR inhibitors. Appl Environ Microbiol 71: 1135–1141. 15746310 10.1128/AEM.71.3.1135-1141.2005 PMC1065175

[42] Altschul SF, Gish W, Miller W, Myers EW, Lipman DJ (1990) Basic local alignment search tool. J Mol Biol 215: 403–410. 2231712 10.1016/S0022-2836(05)80360-2

[43] Pruesse E, Quast C, Knittel K, Fuchs BM, Ludwig W, et al (2007) SILVA: a comprehensive online resource for quality checked and aligned ribosomal RNA sequence data compatible with ARB. Nucleic Acids Res 35: 7188–7196. 17947321 10.1093/nar/gkm864 PMC2175337

[44] Hall TA (1999) BioEdit: a user-friendly biological sequence alignment editor and analysis program for Windows 95/98/NT. Nucl Acids Symp Ser 41: 95–98.

[45] Saitou N, Nei M (1987) The neighbor-joining method: a new method for reconstructing phylogenetic trees. Mol Biol Evol 4: 406–425. 3447015 10.1093/oxfordjournals.molbev.a040454

[46] Tamura K, Peterson D, Peterson N, Stecher G, Nei M, et al (2011) MEGA5: molecular evolutionary genetics analysis using maximum likelihood, evolutionary distance, and maximum parsimony methods. Mol Biol Evol 28: 2731–2739. 21546353 10.1093/molbev/msr121 PMC3203626

[47] Jukes TH, Cantor CR (1969) Evolution of protein molecules. New York: Academic Press.

[48] Felsenstein J (1985) Confidence limits on phylogenies: An approach using the bootstrap. Evolution 39: 783–791. 28561359 10.1111/j.1558-5646.1985.tb00420.x

[49] Nichols GL (2008) Epidemiology. In: Fayer R, Xiao L, editors. *Cryptosporidium* and Cryptosporidiosis. 2nd ed. Boca Raton, FL: CRC Press and IWA Publishing. 79–118.

[50] Caraguel CG, Stryhn H, Gagne N, Dohoo IR, Hammell KL (2011) Selection of a cutoff value for real-time polymerase chain reaction results to fit a diagnostic purpose: analytical and epidemiologic approaches. J Vet Diagn Invest 23: 2–15. 21217022 10.1177/104063871102300102

[51] Xiao L, Ryan UM (2008) Molecular Epidemiology. In: Fayer R, Xiao L, editors. *Cryptosporidium* and Cryptosporidiosis. 2nd ed. Boca Raton, FL: CRC Press and IWA Publishing. 119–171.

[52] Stadhouders R, Pas SD, Anber J, Voermans J, Mes TH, et al (2010) The effect of primer-template mismatches on the detection and quantification of nucleic acids using the 5′ nuclease assay. J Mol Diagn 12: 109–117. 19948821 10.2353/jmoldx.2010.090035 PMC2797725

[53] Chalmers RM, Elwin K, Hadfield SJ, Robinson G (2011) Sporadic human cryptosporidiosis caused by *Cryptosporidium cuniculus*, United Kingdom, 2007–2008. Emerg Infect Dis 17: 536–538. 21392453 10.3201/eid1703.100410 PMC3165992

[54] Robinson G, Elwin K, Chalmers RM (2008) Unusual *Cryptosporidium* genotypes in human cases of diarrhea. Emerg Infect Dis 14: 1800–1802. 18976577 10.3201/eid1411.080239 PMC2630733

[55] Whale AS, Huggett JF, Cowen S, Speirs V, Shaw J, et al (2012) Comparison of microfluidic digital PCR and conventional quantitative PCR for measuring copy number variation. Nucleic Acids Res 40: e82. 22373922 10.1093/nar/gks203 PMC3367212

