# Supplementary material for: The Applicability of TaqMan-Based Quantitative Real-Time PCR Assays for Detecting and Enumerating Cryptosporidium spp. Oocysts in the Environment
Source: PLoS One. 2013 Jun 21;8(6):e66562. doi: 10.1371/journal.pone.0066562 (PMC3689768; doi:10.1371/journal.pone.0066562)
Supplement: Figure S1 — ClustalW alignment of select 18S rRNA sequences with JVA and CRU18S qPCR primers/probes. Species abbreviations: Cpa, Cryptosporidium parvum; Cho, Cryptosporidium hominis; Cca, Cryptosporidium canis; Cmu, Cryptosporidium muris; Cyc, Cyclospora cayetanensis; Ete, Eimeria tenella; Tgo, Toxoplasma gondii. Letters highlighted in grey indicate nucleotide mismatches with the CRU18S primer/probe set. (DOCX) [file pone.0066562.s001.docx]

**Figure S1**

384 394 404 414 424 434 444 454 464 474

....|....|....|....|....|....|....|....|....|....|....|....|....|....|....|....|....|....|....|....|

**Cpa**  **ATGACGGGTAACGGGGAATTAGGGTTCGATTCCGGAGAGGGAGCCTGAGAAACGGCTACCACATCTAAGGAAGGCAGCAGGCGCGCAAATTACCCAATCC**

**Cho**  **ATGACGGGTAACGGGGAATTAGGGTTCGATTCCGGAGAGGGAGCCTGAGAAACGGCTACCACATCTAAGGAAGGCAGCAGGCGCGCAAATTACCCAATCC**

**Cca**  **ATGACGGGTAACGGGGAATTAGGGTTCGATTCCGGAGAGGGAGCCTGAGAAACGGCTACCACATCTAAGGAAGGCAGCAGGCGCGCAAATTACCCAATCC**

**Cmu**  **ATGACGGGTAACGGGGAATTAGGGTTCGATTCCGGAGAGGGAGCCTGAGAAACGGCTACCACATCTAAGGAAGGCAGCAGGCGCGCAAATTACCCAATCC**

**Cyc**  **TTGACGGGTAACGGGGAATTAGGGTTCGATTCCGGAGAGGGAGCCTGAGAAACGGCTACCACATCTAAGGAAGGCAGCAGGCGCGCAAATTACCCAAT--**

**Ete**  **GTGACGGGTAACGGGGAATTAGGGTTCGATTCCGGAGAGGGAGCCTGGGAAACGGCTACCACATCTAAGGAAGGCAGCAGGCGCGCAAATTACCCAAT--**

**Tgo**  **GTGACGGGTAACGGGGAATTAGGGTTCGATTCCGGAGAGGGAGCCTGAGAAACGGCTACCACATCTAAGGAAGGCAGCAGGCGCGCAAATTACCCAATCC**

**JVAF**  **ATGACGGGTAACGGGGAAT**

**JVAP18S**  **.............................................................CATCTAAGGAAGGCAGCAGGCGCG**

484 494 504 514 524 534 544 554 564 574

....|....|....|....|....|....|....|....|....|....|....|....|....|....|....|....|....|....|....|....|

**Cpa**  **TAATA-CAGG---GAGGTAGTGACAAGAAATAACAATACAGGACTTTTTG-GT--TT-T-GTAATTGGAATGAGTTAA-GTAT--AAACCCCTTTACA-A**

**Cho**  **TAATA-CAGG---GAGGTAGTGACAAGAAATAACAATACAGGACTTTTTG-GT--TT-T-GTAATTGGAATGAGTTAA-GTAT--AAACCCCTTTACA-A**

**Cca**  **TAATA-CAGG---GAGGTAGTGACAAGAAATAACAATACAGGACTTTAACAGT--TT-T-GTAATTGGAATGAGTTGA-GTAT--AAACCCCTTTACA-A**

**Cmu**  **TGACA-CAGG---GAGGTAGTGACAAGAAATAACAATACAGGGCCT-AACGGT--CT-T-GTAATTGGAATGAGT-GAAGTAT--AAACCCCTTTACG-A**

**Cyc**  **-GAAAACAGTTTCGAGGTAGTGACGAGAAATAACAATACAGGGCATTTAA--TGCTT-T-GTAATTGGAATGA-T-AG-GAATTTAAAATCCTTC-CAGA**

**Ete**  **-GAAAACAGCTTCGAGGTAGTGACGAGAAATAACAATACAGGGCATTTTA--TGCTT-T-GTAATTGGAATGA-T-GG-GAATGTAAAACCCTTC-CAGA**

**Tgo**  **TGATT-CAGG---GAGGTAGTGACAAGAAATAACAACACTGGAAATTTCA--T--TTCTAGTGATTGGAATGA-T-AG-GAATCCAAACCCCTTT-CAGA**

**CRU18SF**  **.............GAGGTAGTGACAAGAAATAACAATACAGG**

**JVAR**  **........................................GGACTTTTTG-GT--TT-T-GTAATTGG**

584 594 604 614 624 634 644 654 664 674

....|....|....|....|....|....|....|....|....|....|....|....|....|....|....|....|....|....|....|....|

**Cpa**  **GTATCAATTGGAGGGCAAGTCTGGTGCCAGCAGCCGCGGTAATTCCAGCTCCAATAGCGTATATTAAAGTTGTTGCAGTTAAAAAGCTCGTAGTTGGATT**

**Cho**  **GTATCAATTGGAGGGCAAGTCTGGTGCCAGCAGCCGCGGTAATTCCAGCTCCAATAGCGTATATTAAAGTTGTTGCAGTTAAAAAGCTCGTAGTTGGATT**

**Cca**  **GTATCAATTGGAGGGCAAGTCTGGTGCCAGCAGCCGCGGTAATTCCAGCTCCAATAGCGTATATTAAAGTTGTTGCAGTTAAAAAGCTCGTAGTTGGATT**

**Cmu**  **GTATCAATTGGAGGGCAAGTCTGGTGCCAGCAGCCGCGGTAATTCCAGCTCCAATAGCGTATATTAAAGTTGTTGCAGTTAAAAAGCTCGTAGTTGGATT**

**Cyc**  **GTAACAATTGGAGGGCAAGTCTGGTGCCAGCAGCCGCGGTAATTCCAGCTCCAATAGTGTATATTAGAGTTGTTGCAGTTAAAAAGCTCGTAGTTGGATT**

**Ete**  **GTAACAATTGGAGGGCAAGTCTGGTGCCAGCAGCCGCGGTAATTCCAGCTCCAATAGTGTATATTAGAGTTGTTGCAGTTAAAAAGCTCGTAGTTGGATT**

**Tgo**  **GTAACAATTGGAGGGCAAGTCTGGTGCCAGCAGCCGCGGTAATTC-AGCTCCAATAGCGTATATTAAAGTTGTTGCAGTTAAAAAGCTCGTAGTTGGATT**

**CRU18STM** **.....................................................................TTGTTGCAGTTAAAAAGCTCGTA**

684 694 704 714 724 734 744 754 764 774

....|....|....|....|....|....|....|....|....|....|....|....|....|....|....|....|....|....|....|....|

**Cpa**  **TCTG---T-TA-AT-A---ATTTA--T--A-TAA-AATAT-T----TTGATGA---ATAT-TTAT-ATAATAT-TAACATAAT-T-C----A-TA-TTAC**

**Cho**  **TCTG---T-TA-AT-A---ATTTA--T--A-TAA-AATAT-T----TTGATGA---ATAT-TTAT-ATAATAT-TAACATAAT-T-C----A-TA-TTAC**

**Cca**  **TCTG---T-TA-AT-A---ATTTA--T--A-TAT-AATAT-T----T--A--AC--ATAT-TTAT-ATAATAT-TAACATAAT-T-C----A-TA-TTAC**

**Cmu**  **TCTG---T-TGTAT-A---ATCTA--T--A--AT-ATTAC-T-------A--AGGTATATATTAT-AT--TAT-CAACATCCT-T-C----C-TA-TTA-**

**Cyc**  **TCTGTCGTGG---TCATCC-GGCCTTGCCCGT-A-GGG-TGTGCGCCTGG-G-----T-T----GC--CCG--C-GG-CTT-TCTTCCGGTAGC-CTT-C**

**Ete**  **TCTGTCGTGG---TCATCC-GGCGTCGCCCGT-ATGGG-TGTGGGCCTGG-C-----A-T----GC--CCT--C-GG-CTTAT-TTCCGGTAGC-CTT-C**

**Tgo**  **TCTG-C-TGGA-AGCAGCCAGTCC--GCCC-TCA-GGGGTGTGCACTTGGTGA---AT-T-CTAGCATCCT-TCTGG-ATT-TCT-CC---A-CACTT-C**

784 794 804 814 824 834 844

....|....|....|....|....|....|....|....|....|....|....|....|....|....|....|...

**Cpa**  **TA----TA---T-A-----TT----T-T-AG-TATAT-G-A-AATTTTACTTTGAGAAAATTAGAGTGCTTAAAGCAG**

**Cho**  **TA----TT---T-T-----TTT-T-TTT-AG-TATAT-G-A-AATTTTACTTTGAGAAAATTAGAGTGCTTAAAGCAG**

**Cca**  **TA---------T-------TT----A-T-AG-TATAT-G-A-AACTTTACTTTGAGAAAATTAGAGTGCTTAAAGCAG**

**Cmu**  **TA---------T-------TT----C-T-AAATATATAGGA-AACTTTACTTTGAGAAAATTAGAGTGCTTAAAGCAG**

**Cyc**  **-CGCGCTTCGCTGCGTGCGTTGGTGTTCC-G-------G-AAC-TTTTACTTTGAGAAAAATAGAGTGTTTCAAGCAG**

**Ete**  **-CGCGCTTAATTGCGTGTGTTGGTGTTCT-G-------G-AAC-TTTTACTTTGAGAAAGATAGAGTGTTTCAAGCAG**

**Tgo**  **-A----TT-G-TGTG-GAGTTT-T-TTCCAG-------G-A-C-TTTTACTTTGAGAAAATTAGAGTGTTTCAAGCAG**

**CRU18SR**  **.................................................CTTTGAGAAAATTAGAGTGCTTAAAGCAG**
