# Supplementary figures and images for: The Applicability of TaqMan-Based Quantitative Real-Time PCR Assays for Detecting and Enumerating Cryptosporidium spp. Oocysts in the Environment
Source: PLoS One. 2013 Jun 21;8(6):e66562. doi: 10.1371/journal.pone.0066562 (PMC3689768; doi:10.1371/journal.pone.0066562)

**Figure S2**


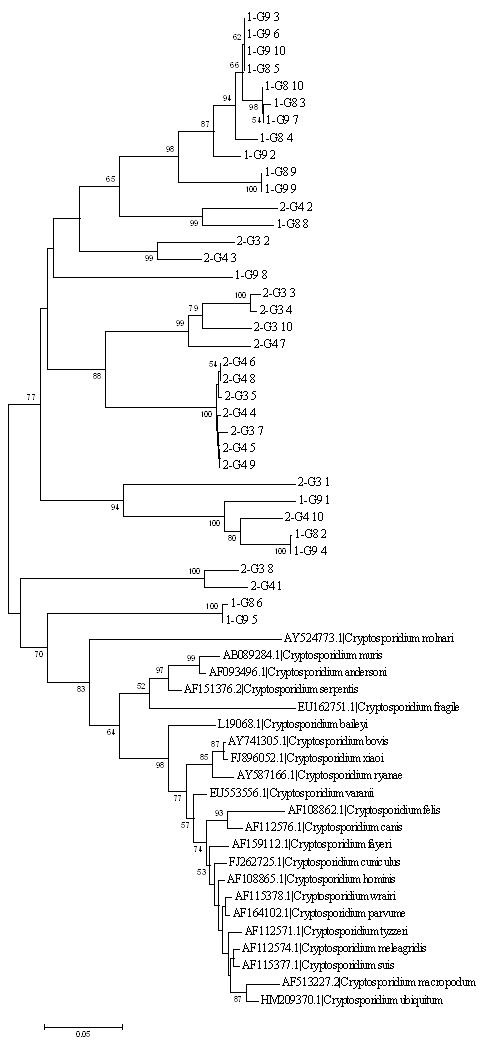

Supplement: Figure S2 — Relationships of 36 environmental sequences to reference Cryptosporidium 18S rRNA sequences. The percentage (>50) of replicate trees in which the associated taxa clustered together in the bootstrap test (500 replicates) are shown next to the branches. The Neighbor-Joining tree is drawn to scale, with branch lengths in the same units as those of the Jukes-Cantor distances used to infer the tree. Branches labeled with 1-G or 2-G prefixes are environmental samples. (JX471018-JX471053). (DOCX) [file pone.0066562.s002.docx]
