# Supplementary material for: The Applicability of TaqMan-Based Quantitative Real-Time PCR Assays for Detecting and Enumerating Cryptosporidium spp. Oocysts in the Environment
Source: PLoS One. 2013 Jun 21;8(6):e66562. doi: 10.1371/journal.pone.0066562 (PMC3689768; doi:10.1371/journal.pone.0066562)
Supplement: Table S1 — BLAST analyses of the 36 environmental sequences obtained from using the CRU18S qPCR primer set1. (DOCX) [file pone.0066562.s003.docx]

**Table S1:**

| **Clones** | **Blast match** | **Length** | **% ID** | **MM** | **Gaps** |
| --- | --- | --- | --- | --- | --- |
| 2-G3_1 | gb\|AY620265.1\|Uncultured cercozoan | 270 | 96.67 | 7 | 2 |
|  | gb\|EU567217.1\|Uncultured cercozoan | 257 | 85.21 | 17 | 21 |
|  | gb\|EF025033.1\|Uncultured eukaryote | 144 | 95.83 | 6 | 0 |
|  | emb\|FN690718.1\| Uncultured Chlorophyta | 146 | 95.21 | 5 | 2 |
|  | gb\|EF025011.1\| Uncultured eukaryote | 146 | 95.21 | 5 | 2 |
| 2-G3_10 | gb\|DQ409123.1\|Uncultured picoplankton | 261 | 99.62 | 1 | 0 |
|  | gb\|FJ765385.1\| Uncultured freshwater eukaryote | 261 | 99.23 | 2 | 0 |
|  | gb\|AY251288.1\|Dinophyceae | 261 | 99.23 | 2 | 0 |
|  | gb\|JF826341.1\| Uncultured marine alveolate | 261 | 98.85 | 2 | 1 |
|  | emb\|FN690253.1\| Uncultured alveolate | 261 | 98.85 | 2 | 1 |
| 2-G3_2 | gb\|EU143989.1\| Uncultured fungus | 261 | 99.62 | 1 | 0 |
|  | gb\|FJ410578.1\| Uncultured fungus | 262 | 92.37 | 18 | 2 |
|  | gb\|FJ410572.1\| Uncultured fungus | 262 | 92.37 | 18 | 2 |
|  | gb\|GU067982.1\| Uncultured fungus | 262 | 91.98 | 19 | 2 |
|  | gb\|AY916724.1\| Marasmius | 268 | 86.57 | 17 | 19 |
| 2-G3_3 | gb\|FJ914495.1\| Uncultured marine dinoflagellate | 260 | 94.62 | 14 | 0 |
|  | gb\|FJ914474.1\| Uncultured marine dinoflagellate | 260 | 94.62 | 14 | 0 |
|  | gb\|EU780651.1\| Ornithocercus magnificus | 260 | 94.62 | 14 | 0 |
|  | gb\|EU780649.1\| Ornithocercus magnificus | 260 | 94.62 | 14 | 0 |
|  | gb\|EU780647.1\| Ornithocercus quadratus | 260 | 94.62 | 14 | 0 |
| 2-G3_4 | gb\|FJ914495.1\| Uncultured marine dinoflagellate | 260 | 95.00 | 13 | 0 |
|  | gb\|FJ914474.1\| Uncultured marine dinoflagellate | 260 | 95.00 | 13 | 0 |
|  | gb\|EU780651.1\| Ornithocercus magnificus | 260 | 95.00 | 13 | 0 |
|  | gb\|EU780649.1\| Ornithocercus magnificus | 260 | 95.00 | 13 | 0 |
|  | gb\|EU780647.1\| Ornithocercus quadratus | 260 | 95.00 | 13 | 0 |
| 2-G3_5 | gb\|FJ410512.1\| Uncultured alveolate | 256 | 94.92 | 9 | 4 |
|  | gb\|FJ410549.1\| Uncultured alveolate | 256 | 94.53 | 10 | 4 |
|  | gb\|DQ244038.1\| Uncultured alveolate | 255 | 94.51 | 12 | 2 |
|  | gb\|FJ410742.1\| Uncultured alveolate | 255 | 94.12 | 13 | 2 |
|  | gb\|FJ410727.1\| Uncultured alveolate | 255 | 94.12 | 13 | 2 |
| 2-G3_7 | gb\|FJ410512.1\| Uncultured alveolate | 256 | 94.53 | 10 | 4 |
|  | gb\|FJ410549.1\| Uncultured alveolate | 256 | 94.14 | 11 | 4 |
|  | gb\|DQ244038.1\| Uncultured alveolate | 255 | 94.12 | 13 | 2 |
|  | gb\|FJ410742.1\| Uncultured alveolate | 255 | 93.73 | 14 | 2 |
|  | gb\|FJ410727.1\| Uncultured alveolate | 255 | 93.73 | 14 | 2 |
| 2-G3_8 | gb\|GQ330636.1\| Uncultured Coccidia | 204 | 93.63 | 11 | 2 |
|  | gb\|AF372780.1\| Uncultured alveolate | 149 | 97.99 | 3 | 0 |
|  | gb\|JF826385.1\| Uncultured marine alveolate | 146 | 95.89 | 6 | 0 |
|  | gb\|JF826379.1\| Uncultured marine alveolate | 146 | 95.89 | 6 | 0 |
|  | emb\|AJ271766.1\| Symbiodinium | 145 | 95.86 | 6 | 0 |
| 2-G4_1 | gb\|EF526764.1\| Uncultured marine eukaryote | 154 | 98.70 | 2 | 0 |
|  | gb\|DQ837534.1\| Gymnodinium dorsalisulcum | 154 | 98.70 | 2 | 0 |
|  | gb\|DQ322643.1\| Crypthecodinium | 153 | 98.69 | 2 | 0 |
|  | gb\|EU780625.1\| Uncultured eukaryote | 165 | 96.36 | 5 | 1 |
|  | gb\|AY664986.1\| Uncultured eukaryote | 164 | 96.34 | 6 | 0 |
| 2-G4_10 | gb\|AY220559.1\| Chlamydomonas monadina | 250 | 99.20 | 2 | 0 |
|  | gb\|AF517096.1\| Chloromonas subdivisa | 250 | 98.00 | 5 | 0 |
|  | gb\|U57694.1\| Chlamydomonas monadina | 250 | 97.20 | 7 | 0 |
|  | emb\|FN690717.1\| Uncultured Chlorophyta | 250 | 97.20 | 7 | 0 |
|  | dbj\|AB001374.1\| Chlamydomonas | 250 | 97.20 | 7 | 0 |
| 2-G4_2 | gb\|DQ898738.1\| Sistotrema oblongisporum | 261 | 99.23 | 2 | 0 |
|  | gb\|DQ898719.1\| Sistotrema oblongisporum | 261 | 99.23 | 2 | 0 |
|  | gb\|AY757263.1\| Sistotrema oblongisporum | 261 | 99.23 | 2 | 0 |
|  | gb\|DQ898715.1\| Sistotrema brinkmannii | 261 | 98.85 | 3 | 0 |
|  | gb\|DQ898712.1\| Sistotrema brinkmannii | 261 | 98.47 | 4 | 0 |
| 2-G4_3 | gb\|EU143989.1\| Uncultured fungus | 254 | 90.94 | 20 | 3 |
|  | gb\|FJ410578.1\| Uncultured fungus | 258 | 90.31 | 22 | 3 |
|  | gb\|FJ410572.1\| Uncultured fungus | 258 | 90.31 | 22 | 3 |
|  | gb\|GU067982.1\| Uncultured fungus | 258 | 89.92 | 23 | 3 |
|  | gb\|AY885046.1\| Uncultured eukaryote | 151 | 96.03 | 5 | 1 |
| 2-G4_4 | gb\|FJ410512.1\| Uncultured alveolate | 256 | 95.70 | 7 | 4 |
|  | gb\|FJ410549.1\| Uncultured alveolate | 256 | 95.31 | 8 | 4 |
|  | gb\|FJ410742.1\| Uncultured alveolate | 255 | 94.90 | 11 | 2 |
|  | gb\|FJ410727.1\| Uncultured alveolate | 255 | 94.90 | 11 | 2 |
|  | gb\|FJ410785.1\| Uncultured alveolate | 255 | 94.51 | 12 | 2 |
| 2-G4_5 | gb\|FJ410512.1\| Uncultured alveolate | 256 | 95.31 | 8 | 4 |
|  | gb\|FJ410549.1\| Uncultured alveolate | 256 | 94.92 | 9 | 4 |
|  | gb\|DQ244038.1\| Uncultured alveolate | 255 | 94.90 | 11 | 2 |
|  | gb\|FJ410742.1\| Uncultured alveolate | 255 | 94.51 | 12 | 2 |
|  | gb\|FJ410727.1\| Uncultured alveolate | 255 | 94.51 | 12 | 2 |
| 2-G4_6 | gb\|FJ410512.1\| Uncultured alveolate | 256 | 95.31 | 8 | 4 |
|  | gb\|FJ410549.1\| Uncultured alveolate | 256 | 94.92 | 9 | 4 |
|  | gb\|FJ410742.1\| Uncultured alveolate | 255 | 94.51 | 12 | 2 |
|  | gb\|FJ410727.1\| Uncultured alveolate | 255 | 94.51 | 12 | 2 |
|  | gb\|FJ410785.1\| Uncultured alveolate | 255 | 94.12 | 13 | 2 |
| 2-G4_7 | gb\|JF826386.1\| Uncultured marine alveolate | 253 | 95.65 | 9 | 2 |
|  | gb\|FJ914416.1\| Uncultured marine dinoflagellate | 252 | 95.63 | 11 | 0 |
|  | gb\|AY664930.1\| Uncultured eukaryote | 257 | 94.94 | 12 | 1 |
|  | dbj\|AB120003.1\| Gyrodinium rubrum | 257 | 94.94 | 12 | 1 |
|  | gb\|JF730800.1\| Uncultured eukaryote | 252 | 95.24 | 12 | 0 |
| 2-G4_8 | gb\|FJ410512.1\| Uncultured alveolate | 256 | 95.31 | 8 | 4 |
|  | gb\|FJ410549.1\| Uncultured alveolate | 256 | 94.92 | 9 | 4 |
|  | gb\|FJ410742.1\| Uncultured alveolate | 255 | 94.51 | 12 | 2 |
|  | gb\|FJ410727.1\| Uncultured alveolate | 255 | 94.51 | 12 | 2 |
|  | gb\|FJ410785.1\| Uncultured alveolate | 255 | 94.12 | 13 | 2 |
| 2-G4_9 | gb\|FJ410512.1\| Uncultured alveolate | 256 | 95.31 | 8 | 4 |
|  | gb\|FJ410549.1\| Uncultured alveolate | 256 | 94.92 | 9 | 4 |
|  | gb\|DQ244038.1\| Uncultured alveolate | 255 | 94.90 | 11 | 2 |
|  | gb\|FJ410742.1\| Uncultured alveolate | 255 | 94.51 | 12 | 2 |
|  | gb\|FJ410727.1\| Uncultured alveolate | 255 | 94.51 | 12 | 2 |
| 1-G8_10 | gb\|HM487049.1\| Uncultured fungus | 251 | 100.00 | 0 | 0 |
|  | gb\|HM486994.1\| Uncultured fungus | 251 | 100.00 | 0 | 0 |
|  | gb\|HM486993.1\| Uncultured fungus | 251 | 100.00 | 0 | 0 |
|  | gb\|HM486989.1\| Uncultured fungus | 251 | 100.00 | 0 | 0 |
|  | gb\|FJ490225.1\| Uncultured eukaryote | 251 | 99.60 | 1 | 0 |
| 1-G8_2 | gb\|GU292343.1\| Ettlia texensis | 259 | 100.00 | 0 | 0 |
|  | dbj\|AB055800.1\| Coelastrella saipanensis | 259 | 100.00 | 0 | 0 |
|  | gb\|GQ375093.1\| Coelastrum astroideum | 259 | 99.61 | 1 | 0 |
|  | dbj\|AB488562.1\| Graesiella emersonii | 259 | 99.61 | 1 | 0 |
|  | gb\|EF024611.1\| Uncultured Scenedesmaceae | 259 | 99.61 | 1 | 0 |
| 1-G8_3 | gb\|HM487049.1\| Uncultured fungus | 251 | 99.60 | 1 | 0 |
|  | gb\|HM486994.1\| Uncultured fungus | 251 | 99.60 | 1 | 0 |
|  | gb\|HM486993.1\| Uncultured fungus | 251 | 99.60 | 1 | 0 |
|  | gb\|HM486989.1\| Uncultured fungus | 251 | 99.60 | 1 | 0 |
|  | gb\|FJ490225.1\| Uncultured eukaryote | 251 | 99.20 | 2 | 0 |
| 1-G8_4 | gb\|FJ687268.2\| Uncultured fungus | 251 | 99.60 | 1 | 0 |
|  | gb\|AY835696.2\| Uncultured eukaryote | 251 | 99.20 | 2 | 0 |
|  | gb\|HM487048.1\| Uncultured fungus | 251 | 98.80 | 3 | 0 |
|  | gb\|HM486995.1\| Uncultured fungus | 251 | 98.80 | 3 | 0 |
|  | gb\|DQ510705.1\| Uncultured fungus | 252 | 98.81 | 2 | 1 |
| 1-G8_5 | gb\|FJ490225.1\| Uncultured eukaryote | 251 | 98.80 | 3 | 0 |
|  | gb\|HM487049.1\| Uncultured fungus | 251 | 98.41 | 4 | 0 |
|  | gb\|HM486994.1\| Uncultured fungus | 251 | 98.41 | 4 | 0 |
|  | gb\|HM486993.1\| Uncultured fungus | 251 | 98.41 | 4 | 0 |
|  | gb\|HM486992.1\| Uncultured fungus | 251 | 98.41 | 4 | 0 |
| 1-G8_6 | gb\|EU709197.1\| Uncultured cercozoan | 270 | 98.89 | 2 | 1 |
|  | gb\|EU709188.1\| Uncultured cercozoan | 270 | 97.41 | 6 | 1 |
|  | gb\|EU709199.1\| Uncultured cercozoan | 270 | 97.04 | 7 | 1 |
|  | gb\|EU709196.1\| Uncultured cercozoan | 270 | 97.04 | 7 | 1 |
|  | gb\|EU709193.1\| Uncultured cercozoan | 271 | 96.68 | 6 | 3 |
| 1-G8_8 | gb\|HQ832429.1\| Pachylepyrium | 262 | 96.56 | 8 | 1 |
|  | gb\|DQ851584.1\| Psilocybe stuntzii | 262 | 96.56 | 8 | 1 |
|  | gb\|AY969186.1\| Uncultured basidiomycete | 262 | 96.56 | 8 | 1 |
|  | gb\|DQ113916.1\| Flammula alnicola | 262 | 96.56 | 8 | 1 |
|  | gb\|HQ827184.1\| Galerina | 262 | 96.18 | 9 | 1 |
| 1-G8_9 | gb\|EF586117.1\| Uncultured eukaryote | 251 | 92.83 | 8 | 10 |
|  | gb\|FJ687268.2\| Uncultured fungus | 255 | 90.98 | 9 | 14 |
|  | gb\|HM487048.1\| Uncultured fungus | 255 | 90.59 | 10 | 14 |
|  | gb\|HM486995.1\| Uncultured fungus | 255 | 90.59 | 10 | 14 |
|  | gb\|DQ510705.1\| Uncultured fungus | 256 | 90.62 | 9 | 15 |
| 1-G9_1 | emb\|AJ781311.1\| Chlamydomonas noctigama | 260 | 100.00 | 0 | 0 |
|  | gb\|AF008241.1\| Chlamydomonas noctigama | 260 | 100.00 | 0 | 0 |
|  | gb\|AF008238.1\| Chlamydomonas noctigama | 260 | 100.00 | 0 | 0 |
|  | gb\|AF008239.1\| Chlamydomonas noctigama | 260 | 99.23 | 2 | 0 |
|  | gb\|AF008242.1\| Chlamydomonas noctigama | 260 | 99.23 | 2 | 0 |
| 1-G9_10 | gb\|FJ490225.1\| Uncultured eukaryote | 251 | 98.80 | 3 | 0 |
|  | gb\|HM487049.1\| Uncultured fungus | 251 | 98.41 | 4 | 0 |
|  | gb\|HM486994.1\| Uncultured fungus | 251 | 98.41 | 4 | 0 |
|  | gb\|HM486993.1\| Uncultured fungus | 251 | 98.41 | 4 | 0 |
|  | gb\|HM486992.1\| Uncultured fungus | 251 | 98.41 | 4 | 0 |
| 1-G9_2 | gb\|AY916573.1\| Uncultured eukaryote | 244 | 96.72 | 6 | 2 |
|  | gb\|FJ490225.1\| Uncultured eukaryote | 233 | 97.85 | 5 | 0 |
|  | gb\|HM487049.1\| Uncultured fungus | 233 | 97.42 | 6 | 0 |
|  | gb\|HM486994.1\| Uncultured fungus | 233 | 97.42 | 6 | 0 |
|  | gb\|HM486993.1\| Uncultured fungus | 233 | 97.42 | 6 | 0 |
| 1-G9_3 | gb\|FJ490225.1\| Uncultured eukaryote | 251 | 98.80 | 3 | 0 |
|  | gb\|HM487049.1\| Uncultured fungus | 251 | 98.41 | 4 | 0 |
|  | gb\|HM486994.1\| Uncultured fungus | 251 | 98.41 | 4 | 0 |
|  | gb\|HM486993.1\| Uncultured fungus | 251 | 98.41 | 4 | 0 |
|  | gb\|HM486992.1\| Uncultured fungus | 251 | 98.41 | 4 | 0 |
| 1-G9_4 | gb\|GU292343.1\| Ettlia texensis | 259 | 100.00 | 0 | 0 |
|  | dbj\|AB055800.1\| Coelastrella saipanensis | 259 | 100.00 | 0 | 0 |
|  | gb\|GQ375093.1\| Coelastrum astroideum | 259 | 99.61 | 1 | 0 |
|  | dbj\|AB488562.1\| Graesiella emersonii | 259 | 99.61 | 1 | 0 |
|  | gb\|EF024611.1\| Uncultured Scenedesmaceae | 259 | 99.61 | 1 | 0 |
| 1-G9_5 | gb\|EU709197.1\| Uncultured cercozoan | 270 | 98.89 | 2 | 1 |
|  | gb\|EU709188.1\| Uncultured cercozoan | 270 | 97.41 | 6 | 1 |
|  | gb\|EU709199.1\| Uncultured cercozoan | 270 | 97.04 | 7 | 1 |
|  | gb\|EU709196.1\| Uncultured cercozoan | 270 | 97.04 | 7 | 1 |
|  | gb\|EU709193.1\| Uncultured cercozoan | 271 | 96.68 | 6 | 3 |
| 1-G9_6 | gb\|FJ490225.1\| Uncultured eukaryote | 251 | 98.80 | 3 | 0 |
|  | gb\|HM487049.1\| Uncultured fungus | 251 | 98.41 | 4 | 0 |
|  | gb\|HM486994.1\| Uncultured fungus | 251 | 98.41 | 4 | 0 |
|  | gb\|HM486993.1\| Uncultured fungus | 251 | 98.41 | 4 | 0 |
|  | gb\|HM486992.1\| Uncultured fungus | 251 | 98.41 | 4 | 0 |
| 1-G9_7 | gb\|HM487049.1\| Uncultured fungus | 251 | 100.00 | 0 | 0 |
|  | gb\|HM486994.1\| Uncultured fungus | 251 | 100.00 | 0 | 0 |
|  | gb\|HM486993.1\| Uncultured fungus | 251 | 100.00 | 0 | 0 |
|  | gb\|HM486989.1\| Uncultured fungus | 251 | 100.00 | 0 | 0 |
|  | gb\|FJ490225.1\| Uncultured eukaryote | 251 | 99.60 | 1 | 0 |
| 1-G9_8 | dbj\|AB616675.1\| Cercozoa | 252 | 92.86 | 14 | 4 |
|  | gb\|FJ790711.1\| Cercomonas | 252 | 92.86 | 14 | 4 |
|  | gb\|AY496048.1\| Neocercomonas jutlandica | 252 | 92.86 | 14 | 4 |
|  | gb\|FJ790755.1\| Uncultured cercomonad | 225 | 94.67 | 12 | 0 |
|  | gb\|FJ790710.1\| Cercomonas celer | 252 | 91.27 | 18 | 4 |
| 1-G9_9 | gb\|EF586117.1\| Uncultured eukaryote | 251 | 92.83 | 8 | 10 |
|  | gb\|FJ687268.2\| Uncultured fungus | 255 | 90.98 | 9 | 14 |
|  | gb\|HM487048.1\| Uncultured fungus | 255 | 90.59 | 10 | 14 |
|  | gb\|HM486995.1\| Uncultured fungus | 255 | 90.59 | 10 | 14 |
|  | gb\|DQ510705.1\| Uncultured fungus | 256 | 90.62 | 9 | 15 |
